# Micro–Macro Coupling Study on the Mechanical Properties of Continuous Fiber-Reinforced Composites

**DOI:** 10.3390/polym16212995

**Published:** 2024-10-25

**Authors:** Na Wang, Zhihua Li, Yubao Peng, Zhuang Jiang, Hongbin Li

**Affiliations:** 1 Hubei Key Laboratory of Modern Manufacturing Quantity Engineering, School of Mechanical Engineering, Hubei University of Technology, Wuhan 430068, China; 4022030026@mails.qust.edu.cn (N.W.); lizhihua127@163.com (Z.L.); 2023031008@mails.qust.edu.cn (Y.P.); jiangz335553@163.com (Z.J.); 2 Department of Electromechanical Engineering, Qingdao University of Science and Technology, Qingdao 266101, China

**Keywords:** micro–macro coupling method, continuous fiber-reinforced composites, molecular dynamics, representative volume element, mechanical properties

## Abstract

As a key and weak point of continuous fiber-reinforced composites (CFRCs), the interface between the fiber and the matrix is vulnerable to failure under external loads, with its performance directly affecting the overall properties of CFRCs. Hence, a micro–macro coupling method that considered the microscopic properties of the interface was utilized to analyze and predict the mechanical properties of CFRCs more accurately. The microscopic mechanical parameters of the fiber–matrix interface, which were obtained using molecular dynamics, were transferred to the representative volume element (RVE). The stiffness matrix of the CFRC, required for the macroscopic finite element model, was then calculated using a unified periodic homogenization method based on the RVE and assigned to the finite element model for a macroscopic simulation. Nylon/continuous carbon fiber specimens were fabricated through additive manufacturing, with the tensile and bending strengths of the specimens obtained through tensile and three-point bending tests. The tensile strength of the experimental specimen was 200.1 MPa, while the result of the simulation containing the interface was 205.5 MPa, indicating a difference of less than 5% between the two. In contrast, the result of the simulation without an interface was 317.7 MPa, representing a high error of 58.7% compared with the experimental results. Moreover, the bending strength, Young’s modulus, and flexural modulus results with and without an interface showed the same trend as that for the tensile strength. This illustrates the effectiveness of the proposed micro–macro coupling method for analyzing and predicting the mechanical properties of CFRCs.

## 1. Introduction

Continuous fiber-reinforced composites (CFRCs), which possess excellent comprehensive mechanical properties, including high specific strength, specific modulus, and design flexibility values, are considered to be the optimal alternatives to traditional metallic materials and are increasingly being valued in various fields, such as the automotive, healthcare, and aerospace industries [1,2,3]. Additive manufacturing (AM) is progressing toward continuous carbon fiber AM, which combines the versatility of AM with the excellent performance of continuous carbon fibers [4]. Owing to its high reliability and the wide range of available shaping materials, fused deposition modeling (FDM), which is a common 3D printing technique, has become the main development direction for producing CFRCs [5]. In the CFRC molding process based on FDM technology, the bonding between the fibers and the matrix is influenced by various parameters, mainly including the layer thickness, heating temperature, fiber volume, etc. [6,7]. When a CFRC is subjected to a load, the interface plays a crucial role in transferring the load from the matrix to the fibers. Therefore, the mechanical properties of the interface directly affect the stiffness and strength of the composite material [6,8,9,10]. Although the interface can be studied at the macroscopic level, understanding the nanoscale interface load transfer at the microscopic level is challenging [8]. Most current CFRC simulation studies model the fibers and matrix separately, and evaluate the overall mechanical properties of the composite material at the macroscopic scale. However, detailed simulation studies of continuous fiber composite materials containing interfaces remain relatively scarce, thus highlighting the need for determining the effective mechanical properties of composite materials using their interfacial parameters.

The mechanical properties of the interface of a composite material are a result of the combined effects of the microstructure and structure, making it difficult to explain the influence of each specific factor through experimental methods. Hence, many researchers have used molecular dynamics (MDs) to elucidate the mechanical properties of composite material interfaces [11,12]. The MDs method, based on the theoretical foundations of statistical and classical mechanics, can provide unique insights into the behavior of nanoscale interfaces, and has been widely applied to study the microscale interfaces of composite materials [13,14]. Through MDs simulations, Zhao et al. [15] demonstrated that adding a 1.8% volume fraction of graphene to polyvinyl alcohol resulted in a ten-fold increase in its Young’s modulus and a 150% increase in its tensile strength. Bao et al. [16] conducted MDs simulations on one- to five-layer graphene sheets under tension, and obtained a Young’s modulus of 1.031 TPa for the monolayer graphene. Gupta et al. [17] obtained a Young’s modulus of 1.290 TPa and Poisson’s ratio of 0.160 for graphene through MDs simulations. However, previous MDs studies have primarily focused on composite materials doped with graphene and carbon nanotubes, whereas the research on CFRCs has mainly used experimental methods to enhance the interface and material properties [18]. As a result, studies utilizing MDs methods to reveal the mechanical properties of CFRC interfaces remain scarce, thus highlighting the urgent need for further research.

Because it is necessary to consider the geometric shapes, material homogeneity, and parameter issues during the macroscopic modeling of a CFRC, introducing the representative volume element (RVE) homogenization method can effectively simplify the model, and link the microscale mechanical properties of a CFRC interface to the macroscopic finite element model [19,20]. The RVE homogenization method involves studying the relationship between the average stress and average strain within a representative cell of a composite material under uniform stress or strain boundary conditions, with numerical simulation methods used to obtain the equivalent mechanical properties of the material. When performing strength and stiffness verification or design optimization on actual engineering structures, inputting these macroscopic constitutive parameters is necessary. Liu et al. [21] utilized the direct equivalent method by selecting an appropriate RVE and combining finite element simulations to analyze the equivalent performance of carbon nanotube-reinforced composite materials. Babu et al. [22] employed RVE models to describe the distribution of short fibers within a composite material, which made it possible to predict the effective stiffness of a short-fiber composite material. Cha et al. [23] developed a multiscale elastoplastic damage model to predict the mechanical performance of a three-dimensional braided composite material. In this model, a sequential multiscale approach was employed to transfer the effective mechanical properties from the microscale to the macroscale. Hadden et al. [24] combined MDs simulations with mechanical simulations at the microscale level to predict the elastic properties of graphene/epoxy resin and graphene/carbon fiber/epoxy resin composite materials. Their predictions were experimentally validated and accurately described the mechanical properties of the composite materials.

The current study builds upon previous research by utilizing MDs methods through Materials Studio 8.0 (MS) software to investigate the mechanical properties of fiber–matrix interfaces. Furthermore, the mechanical parameters of the interface are transferred to the interface of a mesoscopic finite element model (RVE) for homogenization. Subsequently, simulations are conducted using a macroscopic finite element model, and the results are compared with the experimental data. Finally, we investigate the feasibility of verifying the mechanical properties of a CFRC using microscopic MDs and macroscopic finite element simulations.

## 2. Micro–Macro Coupling Method to Analyze the Mechanical Properties of CFRCs

The uncertainty about the internal microstructure and material properties of CFRCs poses challenges to their performance analysis [25]. This paper employs a bottom-up, multiscale modeling approach, starting with detailed simulations at a microscopic scale, to gradually construct performance prediction models at a macroscopic scale, enabling a precise understanding of CFRPs behavior across different scales and predicting their mechanical properties [26,27]. Initially, a molecular dynamics analysis is utilized to investigate the properties of the interfacial region between the fibers and matrix. Subsequently, the molecular-scale analysis results are incorporated into a unidirectional RVE model to characterize the failure strength and characteristic parameters of unidirectional CFRP composites. Finally, based on the outcomes from the RVE model, a homogenized macroscale model is constructed to capture the mechanical properties of CFRPs under various external loading conditions.

### 2.1. Extracting Microscopic Interface Parameters

**Figure 1:**
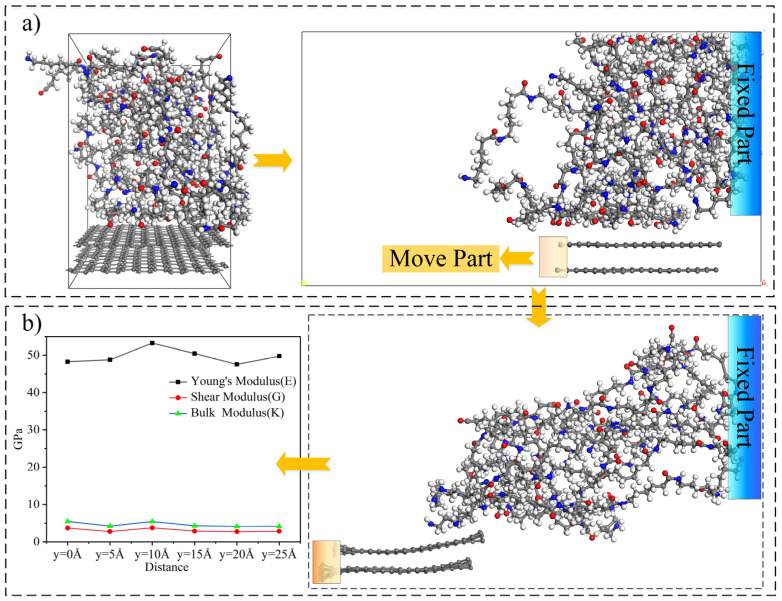
MDs simulation of separation process between carbon fiber and nylon matrix to extract interface’s microscopic parameters. “(**a**)” MDs simulation model and “(**b**)” separation process and interface’s microscopic parameters.

As a critical component of a composite material, the CFRP interface is prone to failure under a load, and its parameters, often at the microscopic level, play crucial roles in the mechanical performance of the composite material. The interface between the fibers and the matrix must be considered to analyze and predict the mechanical properties of a CFRC more accurately. Because the interface is extremely small, almost on a nanoscale, an MDs simulation, which is a powerful tool referenced in the scientific literature [16,17,18], can assist in effectively obtaining its microscopic parameters. MS was used to analyze the interfacial properties of the fiber–matrix interface. An MDs interface model was created. It was 21.3 Å × 30.5 Å × 33.4 Å (where α=β=γ=90°) in size, composed of graphene and nylon 6 molecules, and had a density of 1.15 g/cm^3^. Graphene was selected as the carbon fiber in the model, with the degree of polymerization of the nylon molecular chains determined to be 10. A geometric optimization with 50,000 iteration steps (∆t = 1 fs) was first performed on the established model, followed by a 50 ps dynamic relaxation of the optimized structure in a COMPASS II force field, with a constant volume, temperature, and number of particles (NVT), to release the residual stress and obtain the low-energy conformations [28,29,30]. Next, a standard layer thickness surface in the (100) direction was cut based on the equilibrium structure, followed by adding a 40 Å vacuum layer on the surface as the extraction space for the graphene, as shown in Figure 1a. Based on the established model, a separation simulation of the graphene and nylon 6 matrix was conducted to obtain the interface parameters. During the separation simulation of the carbon fiber and nylon matrix, the molecules at the right end of the nylon 6 matrix were first fixed before moving the graphite layer at the bottom to the left as a whole. Subsequently, a dynamic relaxation of 50 ps was performed on the above model under an NVP ensemble to rebalance the molecules in the model, with a geometric optimization performed on the dynamically relaxed model. Next, the underlying graphene was moved to the left by 5 Å, with a dynamic relaxation and optimization performed again on the modified model, repeating the above steps until the graphene and nylon molecular matrices were completely separated. The separation process is illustrated in Figure 1b.

Based on the interface separation process, the bulk modulus, shear modulus, elastic modulus, and stress–strain relationship functions of the interface were extracted, as listed in Table 1. As illustrated by the interface parameters, minimal variation was observed in the mechanical performance as the extraction distance increased, indicating that the separation distance had almost no effect on the interfacial properties.

At the microscopic scale, the interfacial stress was calculated using the virial stress formula, with the elastic stiffness matrix obtained using the first derivative of the stress with respect to the strain. Although continuous fiber composites are typically approximated as anisotropic materials at the macroscopic level, their interface performance has no directionality at the microscopic level. Therefore, the stiffness matrix of the interface can be assumed to be isotropic.

### 2.2. Homogenization of RVE Modes With and Without Interfaces

Directly assigning the microscopic properties of an interface to a macroscopic analysis model is difficult; moreover, the CFRC exhibited anisotropy on a macroscopic scale. An effective approach to bridge the gap between the micro- and macroscales is to assign the microinterface parameters to the RVE and homogenize the elastic stiffness properties across the different scales, thereby providing estimates of the effective elastic properties. The homogenization method can replace the non-uniform average response of a composite with an equivalent response for a homogenous material. The RVE model, which was first proposed by Hill [31], is defined as the smallest volume element of a material. For this element, the macroscopic constitutive representation is sufficiently accurate to represent the average response. Therefore, the selection and modeling of the RVE should ensure sufficient accuracy. Drugan et al. [32] proposed a method for determining the size of an RVE; this method involves conducting finite element simulations of uniaxial tensile testing using RVEs of different sizes. Comparing the force–displacement curves of RVEs of different sizes makes it possible to observe the influence of the RVE size on the results. Finally, a balance between the model accuracy and complexity is sought to determine the appropriate RVE size. The existing literature has demonstrated that the minimum size of an RVE composite material is 1 mm × 1 mm× 1 mm [21]. The stress–strain curve alignments of the RVE models with the same fiber volume fraction but different sizes were validated during uniaxial tensile simulations, indicating that the 1 mm× 1 mm× 1 mm RVE model was sufficiently accurate. Therefore, this study utilized the same RVE model with a carbon fiber volume fraction of 49% as that in the aforementioned literature. After determining the size of the RVE unit, the arrangement of the fibers in the RVE model was considered. The literature suggests two arrangements of fibers in composite materials: quadrilateral and hexagonal. Based on these arrangements, different unit cell cross-sectional models and grid densities were investigated, and the results showed that the grid density and fiber arrangement had little effect on the effective computational performance. The effective performance of the material mainly depended on the fiber volume fraction. Hence, in this study, a quadrilateral arrangement of fibers was adopted for the RVE model.

**Figure 2:**
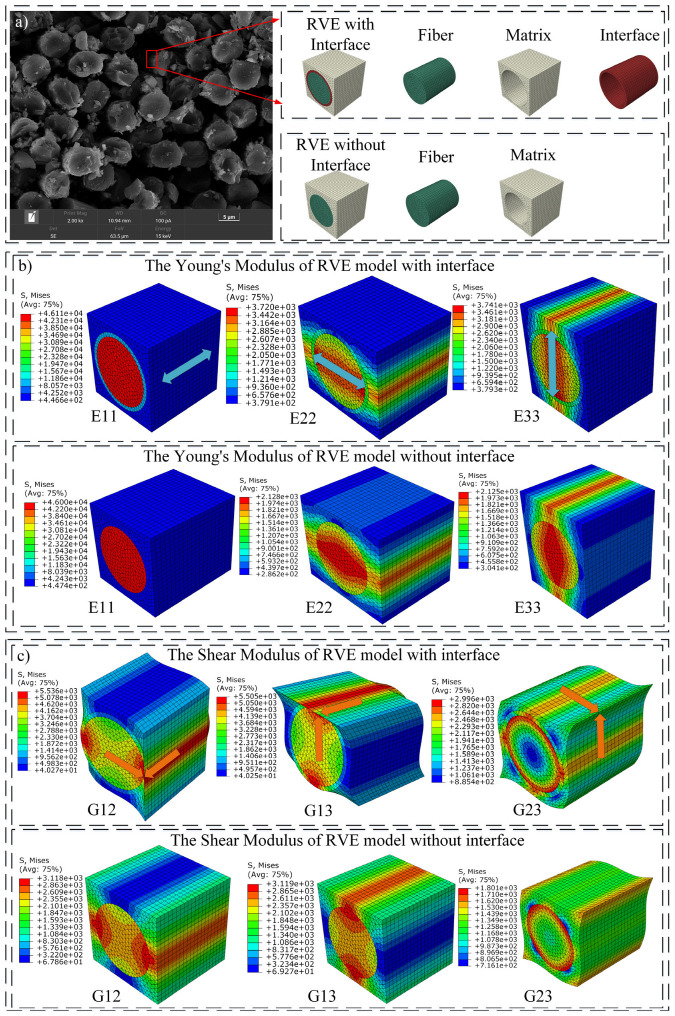
The homogenization of the RVE models. “(**a**)” The RVE models with and without interfaces. “(**b**)” The Young’s modulus of the RVE models with and without interfaces. “(**c**)” The shear modulus of the RVE models with and without interfaces.

After determining the size and fiber arrangement of the RVE model, the RVE model was divided into periodic grids, which on the front and back faces had a mapping relationship to form node pairs to ensure that the equations and displacements could be constrained to obtain the effective elastic properties through subroutines. The appropriate boundary types for the fiber, matrix, and interface contact parts in the RVE model were then separately assigned to ensure the generation of periodic grids. The ABAQUS subroutine EasyPBC was used to estimate the effective elastic properties of the periodic RVE unit by implementing the uniform periodic RVE homogenization method. For comparison, two homogenized RVE models, with and without interfaces, were established to explore the effects of the interfacial properties on the macromechanical properties of the CFRC, as shown in Figure 2a.

After assigning the parameters of the carbon fiber, nylon 6 matrix, and interface for the two RVE models, the homogenized Young’s and shear moduli of the models with and without interfaces were calculated, as shown in Figure 2b,c. The thickness of the interface within the RVE model was determined to be 50 μm, based on the calculations derived from the MDs model. Furthermore, the interface’s Young’s modulus and Poisson ratio were, respectively, specified as 49.66 GPa and 0.3. Table 2 lists the detailed calculation results. According to the results in Table 2, the elastic and shear moduli of the RVE model without an interface are much larger than those of the RVE model with an interface, signifying that the RVE model without an interface has a high stiffness. In the RVE model without an interface, the carbon fiber and nylon matrix at the boundary share nodes, resulting in a rigid connection between them. Consequently, the modulus of the matrix is significantly higher, leading to a larger shear modulus (~30 GPa) for the RVE model without an interface. Upon introducing the interface, the presence of the transition layer brings the modulus of the matrix closer to the normal values, resulting in a shear modulus of 4.9 GPa for the RVE model with an interface, which is consistent with the values reported in reference [33]. After introducing the interface, the properties and thickness of the interface affect the mechanical properties of the RVE model. The interface in the RVE model has a thickness of 50 μm and an elastic modulus of 49.66 GPa. These interface parameters, when coupled with the carbon fiber and resin, result in a Young’s modulus of ~30 GPa for the RVE model with an interface.

### 2.3. Macroscopic Property Calculation of CFRCs Using Homogenized Results of RVE Models

**Figure 3:**
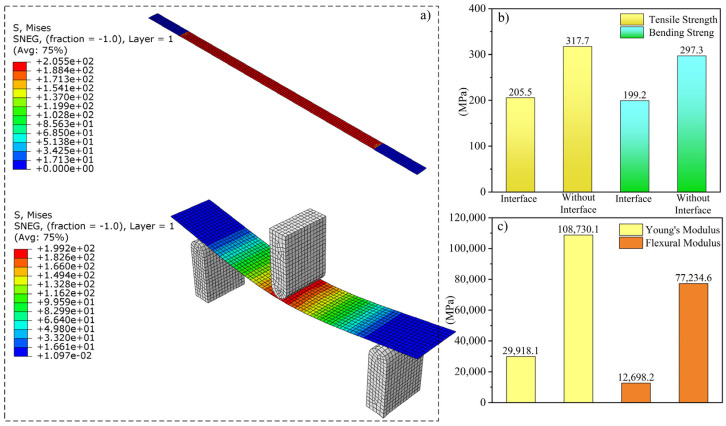
The macroscopic calculation results for the two groups with and without interfaces. “(**a**)” The stretching and bending simulation stress cloud maps. “(**b**)” The tensile and bending strength of the specimens with and without interfaces. “(**c**)” The Young’s modulus and flexural modulus of the specimens with and without interfaces.

The CFRC macromodels for stretching and bending were established in ABAQUS according to GB/T 3354-2014 to perform macro-performance calculations [34,35]. To better fit the actual CFRC products fabricated by FDM, the stretching model consisted of five layers of laminated boards with a thickness of 0.2 mm and a fiber alignment angle of 0° for each layer. Unlike the stretching model, the bending calculation model had 10 layers and a length of 50 mm. After dividing the two models into grids, the homogenized results for the RVE with an interface were assigned to the model, while the homogenized RVE results without interface parameters were assigned to another parallel stretching and bending model. Figure 3 shows the macroscopic calculation results for the two groups with and without interfaces.

According to the calculation results, the tensile strength of the model containing the interface is 205.5 MPa, whereas the benchmark result for the model without interface parameters is 317.7 MPa, or more than 1.5 times that of the model with interface parameters. The bending strength of the model with the interface is 199.2 MPa, and that of the model without the interface is 297.3 MPa. Similar to the tensile strength, the bending strength of the specimen containing the interface is significantly lower than that without the interface. In addition, the moduli of the models coincide with their strength values; the values for the model with the interface are lower than those for the model without the interface. Relevant experiments were conducted to verify the accuracy of the calculation results for the two models.

## 3. Experimental Verification and Discussion

### 3.1. Experimental Tests of CFRC Specimens

PolyMide CoPA, bought from Polymaker, America, is a nylon-based copolymer that possesses excellent strength, heat resistance, and balanced mechanical properties compared to conventional resin matrices. When using PolyMide CoPA to print models, the internal stress within the printed parts can be fully released, resulting in high model stability. PolyMide CoPA is currently the easiest nylon material to use in AM. FDM technology is one of the most convenient AM techniques for combining fibers and polymers owing to its simplicity, low cost, and ease of implementation; hence, FDM was utilized in this study to prepare the PolyMide CoPA/carbon fiber composites. A COMBOT-200 FDM machine from ShanXi Feibo Technology Development Co., Ltd., Xian, China, was used to print the specimens. The materials selected included T3000-1000 (Toray Industries, Inc., Shija, Japan), which has 1000 carbon fibers per bundle, and PolyMide CoPA (the Polymide Company, Shanghai, China). The material parameters are listed in Table 3, which were used in the calculations of the macroscopic performances of the models discussed in Section 2. 

**Figure 4:**
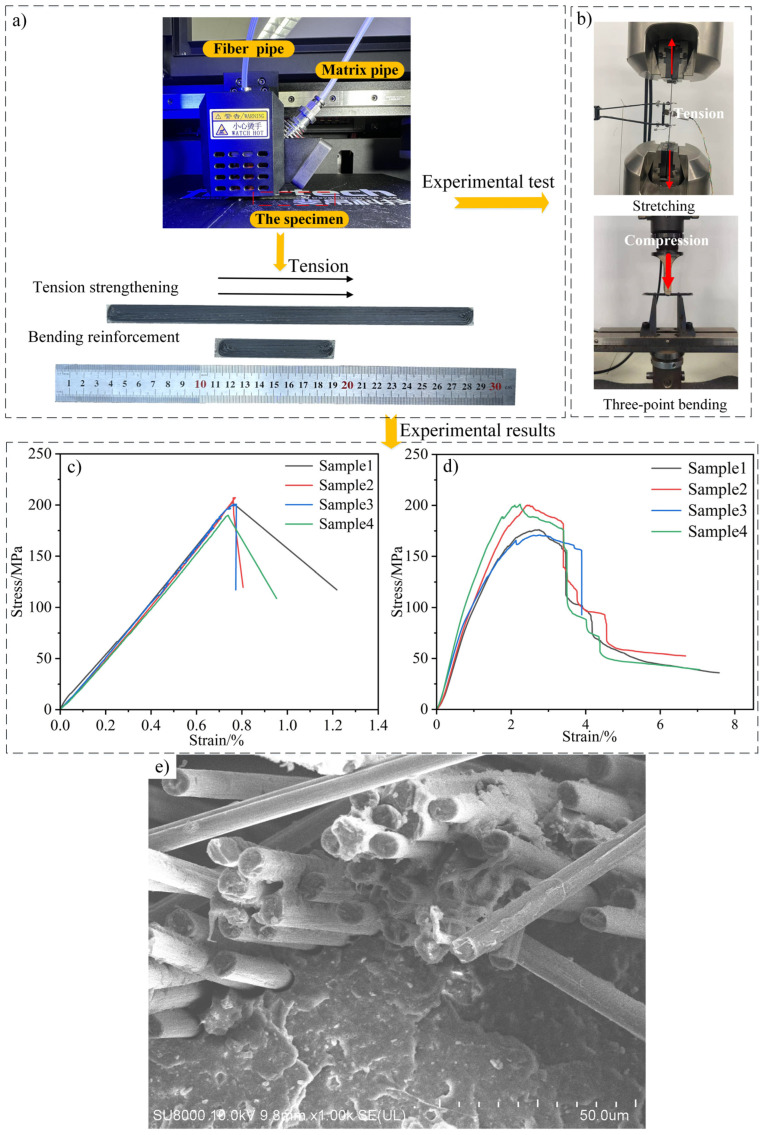
The preparation of the nylon/carbon fiber specimen and the mechanical experiment test. “(**a**)” The tensile and three-point bending experimental specimens fabricated through FDM. “(**b**)” The tensile and three-point bending experiments. “(**c**)” The tensile stress–strain curves. “(**d**)” The three-point bending stress–strain curves. “(**e**)” The scanning electron microscopy of the cross-section of a specimen.

Owing to the influence of the printing parameters, fabricating a CFRC using FDM requires careful consideration [36,37]. Because this study aimed to explore the impact of the interface microstructure on the macroscopic performance of the products, the optimal parameters for preparing the nylon/carbon fiber composites were chosen, as listed in Table 4 [38,39]. To ensure the accuracy of the homogenization results, four uniaxial tensile test specimens of equal rank were prepared according to GB/T 3354-2014 [34] using the same printing parameters; in addition, four bending test specimens were prepared according to GB/T 3356-2014 [35]. Specifically, continuous carbon fibers and PolyMide CoPA feedstock were fed into the heating block through their respective feeders during printing. The PolyMide CoPA feedstock melted because of the action of the heating block, enveloped the fibers, and was extruded from the nozzle to produce the specimens, as illustrated in Figure 4a.

Uniaxial tensile and bending tests were conducted on the CFRP specimens using the same parameters to effectively determine the true mechanical feedback of the material under actual working conditions. The uniaxial tensile and three-point bending experiments on the four specimens are shown in Figure 4. 

### 3.2. Comparison and Discussion

**Figure 5:**
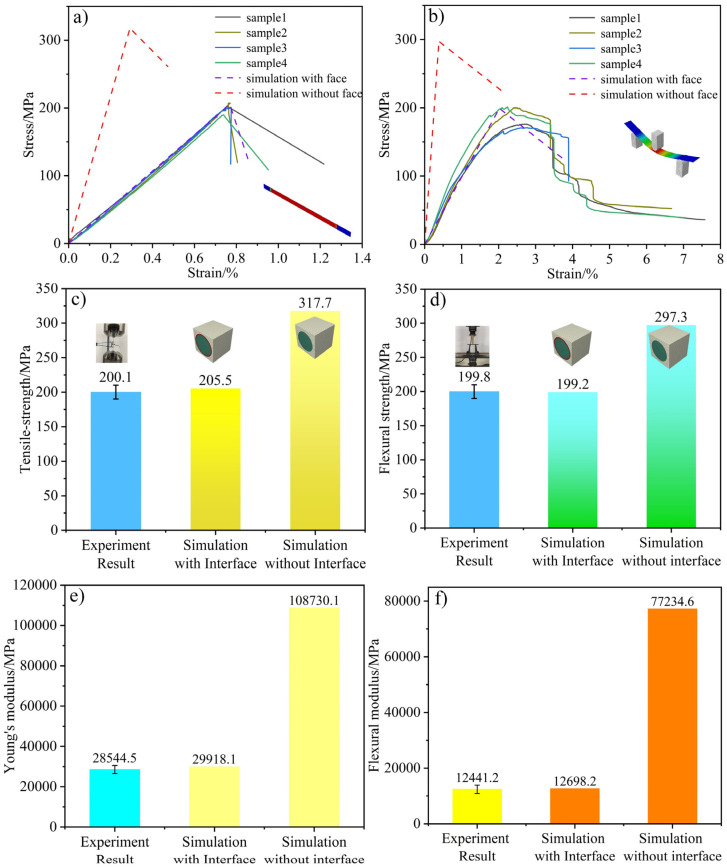
Comparison between simulation and experimental results. “(**a**)” Comparison between the simulation with and without interfaces and experiments of specimens on stretching process. “(**b**)” Comparison between the simulation with and without interfaces and experiments of specimens on bending process. “(**c**)” Comparison between simulation of and experiment on tensile strength. “(**d**)” Comparison between simulation of and experiment on flexural strength. “(**e**)” Comparison between simulation of and experiment on Young’s modulus. “(**f**)” Comparison between simulation of and experiment on flexural modulus.

To more intuitively compare the accuracy of the macroscopic calculation results with and without the interface performance, the calculation and experimental results under the two conditions are presented in the same graph, as shown in Figure 5.

As shown in Figure 5a,b, the stretching and bending finite element simulation processes for the parallel models containing the interface demonstrate a high degree of consistency with the experimental process, with the experimental results being very consistent with the results of the macro-simulations with the interface. The tensile strength of the experimental specimen is 200.1 MPa, while the result of the simulation containing the interface is 205.5 MPa, indicating a difference of less than 5% between the two. In contrast, the result of the simulation without an interface is 317.7 MPa, representing a high error of 58.7% compared with the experimental results. Moreover, the bending strength, Young’s modulus, and flexural modulus results with and without an interface show the same trend as that of the tensile strength, indicating that introducing the interface makes the microstructural RVE model more accurate at predicting the mechanical properties of the composite material. The high consistency between the macroscopic simulations that included the microscopic performance of the interface and the experimental results demonstrates the effectiveness of the micro–macro coupling method for analyzing and predicting CFRC properties. Hence, the results of this study provide new pathways for exploring the fracture and interface failure behaviors of products prepared using AM in the future.

## 4. Conclusions

In this study, the mechanical properties of a CFRC were investigated using a micro–macro coupling method in conjunction with experiments. First, a carbon fiber/nylon MDs model was established in MS, with a simulation of the separation between the carbon fiber and nylon performed to extract the microscopic parameters of the interface between the fiber and the matrix; subsequently, an RVE containing the interface was constructed. The microscopic mechanical parameters of the interface obtained through MDs were assigned to the RVE unit, with the homogenization of the CFRC materials at the mesoscale achieved using this RVE. Finally, the homogenized material properties were assigned to a macroscopic finite element model, with the tensile and bending properties of the macroscopic model calculated using a finite element analysis. Simultaneously, a nylon matrix and carbon fiber reinforcement with the same material properties as those in the macroscopic simulation were used to prepare experimental specimens with the same size as the macroscopic model using FDM technology, and the tensile and bending strengths of the specimens were tested. A comparison between the theoretical simulation and experimental results indicated that the stress–strain relationship of the specimen containing the microscopic interface parameters showed a high level of consistency. Moreover, the theoretically calculated and experimental values of the model containing the interface had an error of no more than 5% in terms of the tensile strength, bending strength, elastic modulus, and bending modulus. However, the macromodel simulation results without the interface differed significantly from the experimental results. These results demonstrate the effectiveness of the micro/macroscopic coupling exploration of CFRC performance conducted in this study, thus providing a theoretical foundation for the controllable preparation and performance prediction of CFRC products.

## Figures and Tables

**Table 1:** The interface performance with the separation distance.

Extraction	Bulk Modulus	Shear Modulus	Young’s Modulus
X = 0 Å	5.4536	3.6828	48.2265
X = 5 Å	4.1948	2.7328	48.7613
X = 10 Å	5.4387	3.7562	53.2489
X = 15 Å	4.2832	2.8534	50.4626
X = 20 Å	4.1047	2.7021	47.5195
X = 25 Å	4.1204	2.8063	49.7416

**Table 2:** Homogenized results of RVE models (E11, E22, E33—Young modulus; G12, G13, G23—shear modulus).

Models	E11	E22	E33	G12	G13	G23	Poisson’s Ratio
With interface (MPa)	30,918.1	12,398.2	12,397.0	4926.5	4921.2	3144.7	0.27
Without interface (MPa)	118,730.1	77,234.6	77,139.2	30,658.1	30,659.1	11,435.5	0.23

**Table 3:** The material parameters.

Mechanical Character	PolyMide CoPA	Continuous Carbon Fiber
Tensile strength (MPA) Young’s modulus (GPA)	50~70 2~3	3520 230
Density (g/cm^3^) Melting point (°C)	1.12 190	1.76 3500

**Table 4:** The parameters for fabricating the CFRC samples.

Printing Settings	Values
Extruder temperature (°C)	260
Platform temperature (°C)	50
Printing speed (mm/s)	2
Layer height (mm)	0.2
Infill ratio	100%

## Data Availability

The original contributions presented in the study are included in the article, further inquiries can be directed to the corresponding author.

## References

[1] Kabir S.F., Mathur K., Seyam A.F.M. (2020). A critical review on 3D printed continuous fiber-reinforced composites: History, mechanism, materials and properties. Compos. Struct..

[2] Katalagarianakis A., Van de Voorde B., Pien N., Polyzos E., Duretek I., Holzer C., Cardon L., Bernaerts K.V., Van Hemelrijck D., Van Vlierberghe S. (2022). The effect of carbon fiber content on physico-mechanical properties of recycled poly (ethylene terephthalate) composites additively manufactured with fused filament fabrication. Addit. Manuf..

[3] Li H., Li Z., Wang N., Peng Y., Jiang Z., Zhang Q. (2023). Improving the mechanical properties of CCFRPLA by enhancing the interface binding energy and strengthening the anti-separation ability of a PLA matrix. Polymers.

[4] Marabello G., Borsellino C., Di Bella G. (2023). Carbon Fiber 3D Printing: Technologies and Performance-A Brief Review. Materials.

[5] Goh G.D., Agarwala S., Goh G.L., Dikshit V., Sing S.L., Yeong W.Y. (2017). Additive manufacturing in unmanned aerial vehicles (UAVs): Challenges and potential. Aerosp. Sci. Technol..

[6] Kong X., Luo J., Luo Q., Li Q., Sun G. (2022). Experimental study on interface failure behavior of 3D printed continuous fiber reinforced composites. Addit. Manuf..

[7] Chacón J.M., Caminero M.A., Núñez P.J., García-Plaza E., García-Moreno I., Reverte J.M. (2019). Additive manufacturing of continuous fibre reinforced thermoplastic composites using fused deposition modelling: Effect of process parameters on mechanical properties. Compos. Sci. Technol..

[8] Wang S., Yan X., Chang B., Liu S., Shao L., Zhang W., Zhu Y., Ding X. (2023). Atomistic Modeling of the Effect of Temperature on Interfacial Properties of 3D-Printed Continuous Carbon Fiber-Reinforced Polyamide 6 Composite: From Processing to Loading. ACS Appl. Mater. Interfaces.

[9] Massarwa E., Tabrizi I.E., Yildiz M. (2021). Mechanical behavior and failure of glass/carbon fiber hybrid composites: Multiscale computational predictions validated by experiments. Compos. Struct..

[10] Mansouri M.R., Fuchs P.F., Baghani M., Schuecker C. (2022). Matrix–fiber interfacial debonding in soft composite materials: Cyclically behavior modeling and microstructural evolution. Compos. Part B Eng..

[11] Sun S., Chen S., Weng X., Shan F., Hu S. (2019). Effect of carbon nanotube addition on the interfacial adhesion between graphene and epoxy: A molecular dynamics simulation. Polymers.

[12] Li M., Zhou H., Zhang Y., Liao Y., Zhou H. (2017). The effect of defects on the interfacial mechanical properties of graphene/epoxy composites. RSC Adv..

[13] Kang H., Zuo K., Wang Z., Zhang L., Liu L., Guo B. (2014). Using a green method to develop graphene oxide/elastomers nanocomposites with combination of high barrier and mechanical performance. Compos. Sci. Technol..

[14] Jensen B.D., Odegard G.M., Kim J.W., Sauti G., Siochi E.J., Wise K.E. (2018). Simulating the effects of carbon nanotube continuity and interfacial bonding on composite strength and stiffness. Compos. Sci. Technol..

[15] Zhao X., Zhang Q., Chen D., Lu P. (2010). Enhanced mechanical properties of graphene-based poly (vinyl alcohol) composites. Macromolecules.

[16] Bao W.X., Zhu C.C., Cui W.Z. (2004). Simulation of Young’s modulus of single-walled carbon nanotubes by molecular dynamics. Phys. B Condens. Matter.

[17] Gupta S., Dharamvir K., Jindal V.K. (2005). Elastic moduli of single-walled carbon nanotubes and their ropes. Phys. Rev. B.

[18] Yan Y., Xu J., Zhu H., Xu Y., Wang M., Wang B., Yang C. (2021). Molecular dynamics simulation of the interface properties of continuous carbon fiber/polyimide composites. Appl. Surf. Sci..

[19] He C., Ge J., Qi D., Gao J., Chen Y., Liang J., Fang D. (2019). A multiscale elasto-plastic damage model for the nonlinear behavior of 3D braided composites. Compos. Sci. Technol..

[20] He C., Ge J., Zhang B., Gao J., Zhong S., Liu W.K., Fang D. (2020). A hierarchical multiscale model for the elastic-plastic damage behavior of 3D braided composites at high temperature. Compos. Sci. Technol..

[21] Liu Y.J., Chen X.L. (2003). Evaluations of the effective material properties of carbon nanotube-based composites using a nanoscale representative volume element. Mech. Mater..

[22] Babu K.P., Mohite P.M., Upadhyay C.S. (2018). Development of an RVE and its stiffness predictions based on mathematical homogenization theory for short fibre composites. Int. J. Solids Struct..

[23] Peng X., Jiang H., Li J., Jia W., Yi B., Wu H., Jiang S. (2024). Generation of two-level representative volume element model for uncertainty analysis of composite materials. Polym. Compos..

[24] Hadden C., Klimek-McDonald D., Pineda E., King J., Reichanadter A., Miskioglu I., Gowtham S., Odegard G. (2015). Mechanical properties of graphene nanoplatelet/carbon fiber/epoxy hybrid composites: Multiscale modeling and experiments. Carbon.

[25] Omairey S.L., Dunning P.D., Sriramula S. (2019). Multiscale surrogate-based framework for reliability analysis of unidirectional FRP composites. Compos. Part B.

[26] Sun Q., Zhou G., Meng Z., Jain M., Su X. (2021). An integrated computational materials engineering framework to analyze the failure behaviors of carbon fiber reinforced polymer composites for lightweight vehicle applications. Compos. Sci. Technol..

[27] Loh J.Y.Y., Yeoh K.M., Raju K., Pham V.N.H., Tan V.B.C., Tay T.E. (2024). A Review of Machine Learning for Progressive Damage Modelling of Fiber-Reinforced Composites. Appl. Compos. Mater..

[28] Hart K.R., Dunn R.M., Sietins J.M., Mock C.M.H., Mackay M.E., Wetzel E.D. (2018). Increased fracture toughness of additively manufactured amorphous thermoplastics via thermal annealing. Polymer.

[29] Jain P.A.K., Sattar S., Mulqueen D., Pedrazzoli D., Kravchenko S., Kravchenko O. (2022). Role of annealing and isostatic compaction on mechanical properties of 3D printed short glass fiber nylon composites. Addit. Manuf..

[30] Li H., Jiang Z., Zhang Q., Zhang H., Zhang J. (2023). Improving the mechanical property of continuous fibre reinforced composites by promoting the polymer molecular chain activity. Compos. Commun..

[31] Hill R. (1963). Elastic properties of reinforced solids: Some theoretical principles. J. Mech. Phys. Solids.

[32] Drugan W.J., Willis J.R. (1996). A micromechanics-based nonlocal constitutive equation and estimates of representative volume element size for elastic composites. J. Mech. Phys. Solids.

[33] Barile C., Kannan V.P., Locasale A., Casavola C. (2023). About Shear Properties of Plain Weave Fabric CFRP at High Temperatures: Analytical and Experimental Approaches. Appl. Compos. Mater..

[34] (2014). Test Method for Tensile Properties of Oriented Fiber Reinforced Polymer Matrix Composites.

[35] (2014). Test Method for Bending Properties of Oriented Fiber Reinforced Polymer Matrix Composites.

[36] Mohamed Y.S., Abdelbary A. (2023). Theoretical and experimental study on the influence of fiber orientation on the tensile properties of unidirectional carbon fiber/epoxy composite. Alex. Eng. J..

[37] Kousiatza C., Tzetzis D., Karalekas D. (2019). In-situ characterization of 3D printed continuous fiber reinforced composites: A methodological study using fiber Bragg grating sensors. Compos. Sci. Technol..

[38] Islam M.N., Baxevanakis K.P., Silberschmidt V.V. (2023). Viscoelastic characterisation of additively manufactured composites with nylon matrix: Effects of type and orientation of fibres. Compos. Part B.

[39] Venkatesh R., Britto J.J.J., Amudhan K., Anbumalar V., Prabhakaran R., Sakthi R.T. (2023). Experimental investigation of mechanical properties on CF reinforced PLA, ABS and Nylon composite part. Mater. Today Proc..

